# Mitochondrial genome data confirm that yaks can serve as the intermediate host of *Echinococcus canadensis* (G10) on the Tibetan Plateau

**DOI:** 10.1186/s13071-018-2684-0

**Published:** 2018-03-09

**Authors:** Yantao Wu, Li Li, Guoqiang Zhu, Wenhui Li, Nianzhang Zhang, Shuangnan Li, Gang Yao, Wenjun Tian, Baoquan Fu, Hong Yin, Xingquan Zhu, Hongbin Yan, Wanzhong Jia

**Affiliations:** 1 State Key Laboratory of Veterinary Etiological Biology/Key Laboratory of Veterinary Parasitology of Gansu Province/Lanzhou Veterinary Research Institute, Chinese Academy of Agricultural Sciences, Lanzhou, 730046 Gansu Province People’s Republic of China; 2 Jiangsu Co-innovation Center for Prevention and Control of Important Animal Infectious Disease, Yangzhou, 225009 Jiangsu Province People’s Republic of China

**Keywords:** *Echinococcus canadensis*, G10 genotype, Yak, Mitochondrial genome

## Abstract

**Background:**

Cervids used to be considered the only animal intermediate hosts of the G10 genotype of *Echinococcus canadensis.* Yaks are often herded in the Qinghai-Tibet Plateau, China, where echinococcosis remains prevalent. However, no *E. canadensis* G10 cases have been recorded in yaks until now. The aim of our study was to identify causative agents of echinococcosis in yaks in this region.

**Methods:**

Total genomic DNA was extracted from the germinal layer of one hydatid using a Blood and Tissue Kit. Full-length mitochondrial (mt) cytochrome *c* oxidase subunit 1 (*cox*1) and NADH dehydrogenase subunit 1 (*nad*1) genes were amplified by PCR. All purified PCR products were directly sequenced in both directions. Then seven pairs of overlap primers were designed to amplify the entire mt genome sequence of a suspected *E. canadensis* G10 isolate. Phylogenetic analyses were performed based on concatenated nucleotides from the 12 protein-coding genes of mt genomes of *Echinococcus* species in a Bayesian framework using MrBayes v3.1 and implementing the GTR + I + G model.

**Results:**

Hydatids were found in yaks (*n* = 129) when organs were inspected at the slaughterhouse in Maqu county, Gannan Tibetan Autonomous Prefecture, Gansu Province, China in October 2016. Of these, 33 (25.6%) harbored up to a dozen hydatid cysts. One cyst from each yak was characterized by sequencing its mitochondrial (mt) *cox*1 and *nad*1 genes. On the basis of these sequence data, 32 cysts were identified as *Echinococcus granulosus* (*sensu stricto*) (G1-G3) and the remaining one was identified as the G10 genotype of *E. canadensis*. Its mt genome was then fully sequenced and compared with that of the G10 genotype in GenBank (AB745463). Phylogenetic analysis using complete mt genomes confirmed the Chinese cyst as belonging to the G10 genotype.

**Conclusions:**

To our knowledge, this is the first report globally of *E. canadensis* (G10) from yaks in China, which suggests that the G10 genotype has a wider geographical distribution and broader host range than previously believed. This genotype has therefore potential risks to human health and animal husbandry.

## Background

Cystic echinococcosis (CE) is one of the most important parasitic diseases in humans and one of the 17 neglected diseases (NTDs) prioritized by the World Health Organisation (WHO) in 2012. It is a widespread zoonosis caused by the cyst stage of *Echinococcus granulosus* (*sensu lato*) [1, 2]. Echinococcosis or hydatidosis affects various internal organs of terrestrial mammals including humans, livestock and wildlife [3].

Recent phylogenetic studies based on both mitochondrial and nuclear DNA genes show that *E. granulosus* (*s.l.*) is comprised of at least five independent species: *E. granulosus* (*s.s.*) (genotypes G1-G3), *E. equinus* (G4), *E. ortleppi* (G5), *E. canadensis* (G6-G10) and *E. felidis* [3–6]. Molecular and morphological studies suggest that it would be better if these *E. granulosus* (G6-G10) be re-classified as a separate species (i.e. *E. canadensis*) [5, 7–10]. These *E. canadensis* genotypes closely match the intermediate host-associated strains described in the earlier reports where *E. canadensis* G10 was named as cervid strain [11], which was first found in cervids in northeastern Finland representing a distinct genotype [12]. Cervids used to be thought the only animal intermediate host of G10 genotype of *E. canadensis*. However, a human case of the G10 type was recently reported in China [2].

Its milk, meat, dung and wool make the yak important for native herdsmen on the Qinghai-Tibet Plateau, where echinococcosis remains prevalent [13–16]. It has been reported that only *E. granulosus* (*s.s.*) and *E. canadensis* (G6) were observed in yaks [17–21]. Nevertheless, no *E. canadensis* G10 cases have been recorded in yaks until now. The aim of our study was to identify the causative agents of echinococcosis in yaks in this region. We characterized yak-derived CE isolates by sequencing selected mitochondrial (mt) genes or the entire mt genome and speculated on possible transmission routes of CE.

## Methods

From August to October each year, many yaks and Tibetan sheep from grazing areas on the Qinghai-Tibet Plateau are slaughtered for human consumption at local slaughterhouses. We collected CE cyst specimens from yaks at a slaughterhouse near Maqu City in Maqu County, Gannan Tibetan Autonomous Prefecture, Gansu Province, China, in October 2016. The slaughterhouse (33^o^59'30"N, 102^o^4'7"E; 3490 m above sea level) is situated near the eastern end of the plateau.

The surface of each cyst was cleaned with 75% alcohol cotton balls. Then, in the ultra-clean bench, the endocyst was repeatedly washed out with a phosphate buffer solution and the washings transferred to 1.5 ml sterile centrifuge tubes using a sterile syringe. The tubes were centrifuged at 3000× *g* for 10 min at room temperature. After the supernatant was poured out, a wet preparation of the sediment was examined for the presence of protoscolices under a microscope. Total genomic DNA was extracted from the germinal layer of the cyst using a Qiagen Blood and Tissue Kit (Qiagen, Hilden, Germany) according to the manufacturer’s instructions and eluted into 100 μl H_2_O, followed by RNase treatment step, and was stored at -20 °C until use.

Full-length *cox*1 (~1853 bp) and *nad*1 (~1286 bp) genes were amplified using the primer pairs 5′-GAA AAT TGT GGA GTT ACT GCT-3′ / 5′-AAG CAT GAT GCA AAA GGC AAA TAA ACC-3′ for the *cox*1 and 5′-ATT ATA GAA AAT TTT CGT TTT ACA CGC-3′ / 5′-ATT CAC AAT TTA CTA TAT CAA AGT AAC C-3′ for the *nad*1. Cycling parameters for both were as follows: an initial denaturation step at 94 °C for 4 min, 35 cycles at 98 °C for 15 s, 52–55 °C for 30 s, and 72 °C for 2 min, followed by a final extension step at 72 °C for 10 min. Each PCR reaction yielded a single band detected in a 1.0% (*w*/*v*) agarose gel stained with GelRed. Each PCR product was purified for sequencing by gel-cut and DNA was recovered through a column according to the manufacturer’s instructions (AxyPrep DNA Gel Extraction Kit by AxyGen, Suzhou, China). All purified PCR products were directly sequenced in both directions using Sanger dideoxy chain termination in an ABI 3730 DNA sequencer at Sangon Company (Shanghai, China). The PCR primers were used as sequencing primers. All the raw sequences were assembled using the software package Chromas, edited and blasted online (https://blast.ncbi.nlm.nih.gov/Blast.cgi?PAGE_TYPE=BlastSearch) to determine the *Echinococcus* species or genotype of each cyst sample.

A single complete mt genome was sequenced to further confirm whether a cyst (~2 cm in diameter) (Specimen 1) belonged to the G10 genotype of *E. canadensis*. Seven pairs of oligonucleotide primers were designed based on the conserved regions from published complete mtDNA sequences of *E. shiquicus*, *E. multilocularis*, *E. equinus*, *E. ortleppi*, *E. granulosus* (*s.s.*) (G1-G3), *E. granulosus* (*s.l.*) (G6-G10) (Tables 1 and 2). The overlapping PCR products amplified by these primers, ranging from 1885 bp to 2622 bp in length, covered the entire mt genome of Specimen 1. PCR reactions were carried out using a standard 3-step regime: 94 °C for 4 min (initial denaturation), 35 cycles of 98 °C for 30 s (denaturation), 52–56 °C for 30 s (annealing), 72 °C for 3 min (extension), followed by a final hold at 72 °C for 10 min. Sequencing and assembly were as above. The mt genome sequence was annotated through alignment with the complete mtDNA sequence of *E. canadensis* (G10) (AB745463).

**Table 1:** The mitochondrial genomes of *Echinococcus* spp. used for inference of the phylogenetic tree

Genotype/species	Size (bp)	Host	GenBank ID	Origin
G1/*E. granulosus* (*s.s.*)	13,588	Sheep	AF297617	UK
G4/*E. equinus*	13,598	Horse	AF346403	UK
G5/*E. ortleppi*	13,717	Cattle	AB235846	Argentina
G6/*E. canadensis*	13,721	Camel	AB208063	Kazakhstan
G7/*E. canadensis*	13,719	Pig	AB235847	Poland
G8/*E. canadensis*	13,717	Moose	AB235848	USA
G10/*E. canadensis*	13,720	Moose	AB745463	Finland
*E. felidis*	13,632	Lion	NC021144	Uganda
*E. multilocularis*	13,738	Vole	AB018440	Japan
*E. oligarthrus*	13,791	Laboratory mice	AB208545	Panama
*E. shiquicus*	13,807	*O. curzoniae*	AB208064	China
*E. vogeli*	13,791	Unknown	AB208546	Colombia
*T. solium*	13,709	Pig	AB086256	China
G10/*E. canadensis* Yak GS	13,603	Yak	MG597240	China (this study)

**Table 2:** Primers for amplification of seven overlapping DNA fragments and their positions in the mtDNA of *Echinococcus* (G10) isolate from Gansu, China, and four additional primer pairs used to amplify a region containing SNR. The positions of the primers are based on the mt genome sequence of *E. canadensis* (G10; AB745463)

Primer name	Primer sequence (5′ → 3′)	Positions on the H-strand	Size of PCR product (bp)
F1	TTTGTAAAGATGCCAGAAAA	244–266	2110
R1	AYCTAGATCATTTTTTTGGA	2337–2356	
F2	GCCCCATATATGTATAGTAT	2225–2244	1962
R2	TATACACCGAAGAATAGCAT	3897–3916	
F3	GATTTRGTGTATTTTCATTCRTA	3710–3732	2508
R3	CCAAAACACCCTAACCTAATAT	6196–6217	
F4	ATCGTTTGCCWTATTGTTATAG	5965–5986	2622
R4	TAACGGAAAATAAATTCACA	8567–8586	
F5	TGCTGTTAACTTCAAGAAATGG	8418–8439	1885
R5	ACATAACATAATGAAAATGAGC	10,281–10,302	
F6	ATATGTTTACTGTTGGGTTRGAT	10,027–10,049	2352
R6	GCAGCACATAGACTTGGCTT	12,360–12,379	
F7	CATCTGCGGTTARTCTGTTTTC	12,036–12,057	2143
R7	TAATGCTTAAAACTAACTCATA	437–458	
F8	TTTATTTTTGTGTCGGTGTTTG	13,448–13,469	1387
R8	CCCGCATAGCCTCCAACAA	1096–1114	
F9	TTCTGGTGTTAAGTGTTGTG	13,659–13,678	498
R9	TAACTTCTGACATAGCTACC	417–436	
F10	GGCTTGTGTGTATTATTTGG	13,514–13,533	793
R10	ACAAACCTATACTAACACAC	567–586	
F11	GGTGTTAAGTGTTGTGGCCAGAAA	13,663–13,686	530
R11	GAAACATCCATAATTAATGCTTAAAACTAACTC	440–472	

*Abbreviations*: R, A/G; W, A/T; Y, T/C

Phylogenetic analyses were performed using concatenated nucleotides from the 12 protein-coding genes of the mt genomes of *E. canadensis* (G10) and other *Echinococcus* species (Table 1) in a Bayesian framework using MrBayes v.3.1 [22] and implementing the GTR + I + G model of protein coding genes evolution as described previously [23]. The mt genome sequence of *Taenia solium* was used as the outgroup. MrBayes settings were lset nst = 6, rates = invgamma; two chains (temp = 0.2) were run for 1,000,000 generations and sampled every 1000 generations. Convergence was assessed using Tracer v.1.4 [24], with a discarded 'burn-in' period of 1000 trees. Nodal support was expressed using posterior probabilities.

## Results

Of 129 yaks examined, 33 (25.6%) harbored hydatid cysts. Thirty two cyst samples were identified as *Echinococcus granulosus* (*s.s.*) (G1-G3), while Specimen 1 was identified as belonging to the G10 genotype of *E. canadensis* (here designated Yak-GS). Our Specimen 1 (G10, Yak-GS), found near the right lobe margin of the yak’s lung, contained protoscolices in hydatid fluid. The *cox*1 and *nad*1 full-length gene sequences of this cyst showed a respective 99.75% and 99.36% similarity with those (GenBank: KJ663947 and KJ663949) from a 66-year-old female CE patient in northeastern China. Of the two base substitutions in the *cox*1 gene, one, at position 425 (G to A), produced an amino acid change from tyrosine to cysteine. Of the five substitutions in the *nad*1 gene, one, at position 522 (A to G), caused an amino acid change from cysteine to tryptophan (Table 3).

**Table 3:** Differences in nucleotides and amino acids at the *cox1* and *nad1* loci of *E. canadensis* G10 between the sequences from Yak_GS (MG597240) and a cystic echinococcosis patient of NE China (KJ663947)

Gene	GenBank ID	No. of mutations (% similarity)	Codon (amino acid)/Nucleotide position^a^
*cox*1	KJ663947	2 (99.75)	TAT (Y)/425; GGA(G)/888
	MG597240		TGT (C)/425; GGG(G)/888
*nad*1	KJ663949	5 (99.36)	GGC (G)/117; AGC (S)/207; GGT (G)/231; GTG (V)/315; TGG (W)/522
	MG597240		GGT (G)/117; AGT (S)/207; GGC (G)/231; GTA (V)/315; TGT (C)/522

*Abbreviations*: *Y* tyrosine, *G* glycine, *C* cysteine, *S* serine, *V* valine, *W* tryptophan

^a^Nucleotide position numbers based on AB745463, with the beginnings of the coding region of the *cox*1 and *nad*1 loci as position no. 1, respectively

The mt genome of G10 (Yak-GS) (MG597240) was 13,603 bp in length and A + T-rich (67.67%). The mt genome sequence of G10 (Yak-GS) had 99.6% identity with that of the previously reported cervid strain (13,720 bp in length): the main difference was less 117 bp from the sequence (GenBank: AB745463) occurred in the short non-coding region (SNR) located between tRNA-Tyr and tRNA-Leu. In order to further confirm the difference, four more primer pairs (F8-R8 to F11-R11 in Table 2) were used to amplify a region containing SNR.

**Fig. 1:**
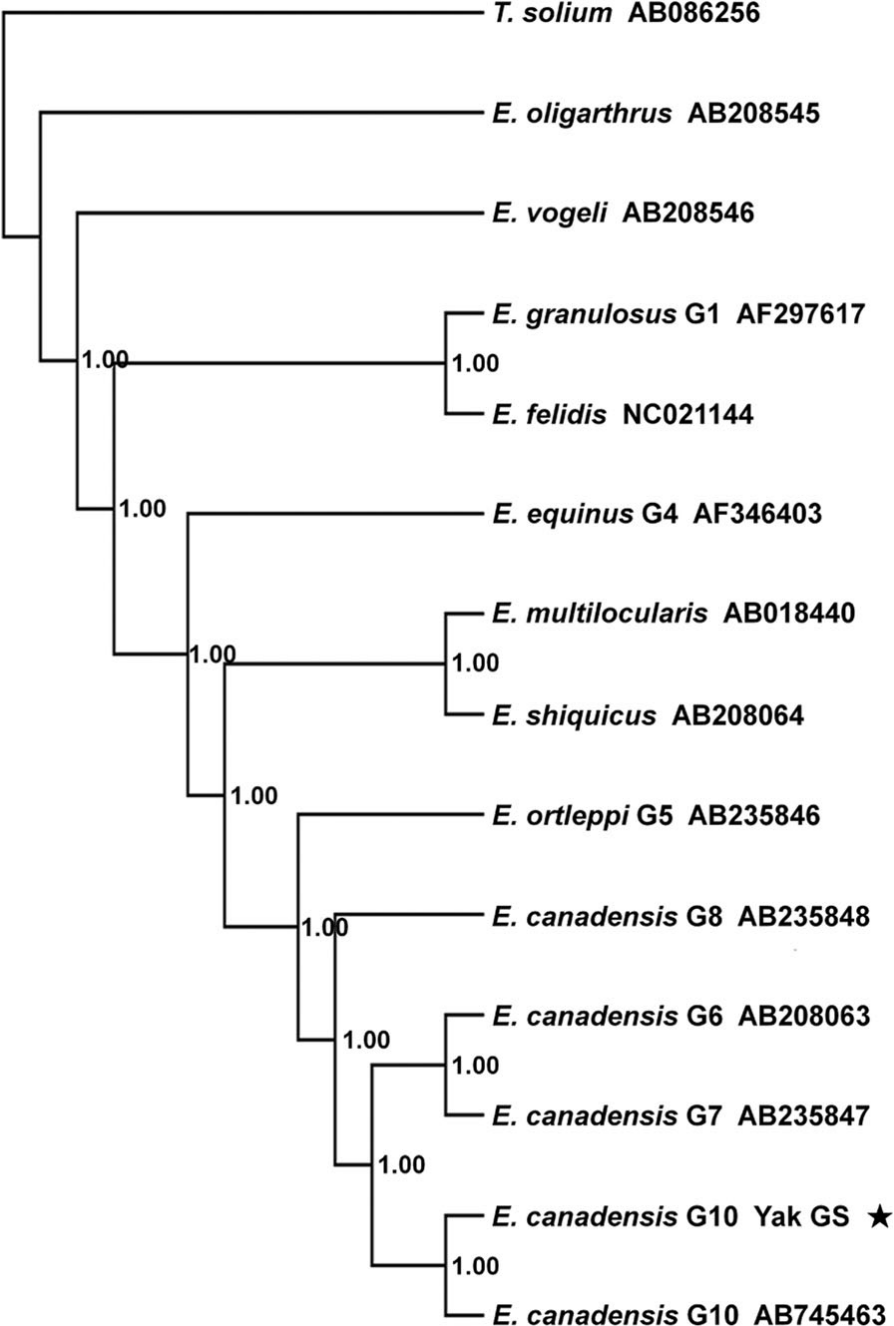
Phylogenetic relationships of species of *Echinococcus* (Taeniidae) estimated from mtDNA protein-coding genes using a Bayesian analysis of concatenated nucleotides. Three isolates of *E. canadensis* are included to indicate within-species variation. Nodal support is indicated by posterior probabilities. The scale-bar indicates the number of substitutions per site

The Bayesian tree inferred from concatenated nucleotides of the 12 protein-coding genes of the mt genomes is shown in Fig. 1. This tree demonstrated strong support for each species or genotype of *Echinococcus*. The Yak-GS sequence was a sister to *E. canadensis* G10 (GenBank: AB745463) with posterior support value of 100%. This confirms Yak-GS as belonging to *E. canadensis* G10.

## Discussion

*Echinococcus canadensis* (G10) was first discovered and named in northeastern Finland based on five isolates of cervid origin [12, 25]. Adults and larvae of *E. canadensis* G10 were identified in wolves and dogs as definitive hosts, and three deer species (reindeer, moose and elk) as intermediate hosts, using molecular genetic techniques [2, 3, 8, 9, 12, 26–34]. The wolf as the typical definitive host of *E. canadensis* (G10) is an opportunity predator, and the availability of prey and the degree of difficulty in its acquisition determine the diet composition [35]. Thus, wolves have different diets in the different regions [36]. The typical life-cycle of this genotype was “wolf-cervid”, which has been clarified using molecular methods in Estonia, Mongolia, USA, Canada, Finland, Sweden and Russia [3, 8, 9, 12, 28–33, 37, 38]. Wolves on the Tibetan Plateau mainly prey on hares, yaks and small rodents in the plant green period, and mainly prey on yaks, sheep and hares in the plant withering period [36, 39]. There are many species of cervids living on the Tibetan Plateau, such as the white-lipped deer (*Gervus albirostris*), red deer (*Cervus elaphus*), Fea’s muntjac (*Muntiacus feae*), etc. [40, 41]. Until now, however, there was no report on the infection of *Echinococcus* species in cervids, and whether the wolf-cervid cycle exists in this region and needs further study. In addition, a “dog-cervid” life-cycle has been reported in Canada and Finland where dogs had access to offal and carcasses [8, 29–32, 34, 37].

In recent years, *E. canadensis* (G10) has been reported in humans in three countries: Mongolia (2010, 2013), Russia (2014) and Finland (2015) [26–28, 34]. In 2014, a cyst from a 66-year-old female CE patient in Northeastern China’s Heilongjiang Province was also identified as *E. canadensis* (G10) using *cox*1 and *nad*1 genes. This was the first report of the G10 genotype of *E. canadensis* from humans in China. However, the life-cycle of the G10 genotype in this area remains unknown [2]. The *cox*1 gene sequence from this patient (GenBank: KJ663947) was identical with those from wolves in Mongolia, suggesting involvement of this species in China. We have not identified any dogs infected with G10 in China in studies carried out at our laboratory in recent years [2]. However, a “dog-livestock” life-cycle of the G10 genotype cannot be ruled out in Gannan Tibetan Autonomous Prefecture or neighboring regions.

The yak (*Bos grunniens*), a bovid species, inhabits steppes of the Himalayan highlands and was domesticated on the Tibetan plateau about 3000 years ago [42]. More than 14 million domestic yaks live on the Qinghai-Tibetan Plateau in China, accounting for about 95% of the world yak population [15, 43, 44]. The native people totally depend on their yaks herd to support their livelihood [14]. Due to the physical environment and socio-economic situation, herdsmen in this locality classified as ‘semi-nomadic’ practice a nomadic lifestyle for most months of the year and yaks graze only on natural pasture throughout the year and are not offered supplements [43, 45, 46]. Local herdsmen reside in permanent dwellings usually within or close to large settlements during the winter, while they move yaks to summer pastureland where there are no permanent settlements in spring [45]. Dogs are also important to protect herders and their livestock. Influenced by local customs, free-roaming dogs are very commonly seen in Tibetan areas, even in urban places like Lhasa, Yushu and other cities. The native Tibetan pastoralists tend to kill and process domesticated livestock themselves [14, 47]. The fresh internal organs (offal) of yaks and sheep are discarded carelessly, often being eaten by dogs [14, 48], which undoubtedly increases the risk of transmission of hydatid disease. Therefore, in these areas humans probably acquire hydatid infection mostly through the yak-dog cycle. In order to prevent the transmission of echinococcosis in this region, we strongly suggest better control and management of both family dogs and stray dog populations, improvement of slaughter hygiene management with more careful inspection and handling of offal.

Currently, wolves and dogs are known to be the definitive hosts, and cervids (moose, elk and reindeer) are generally considered the only animal intermediate hosts of *E. canadensis* (G10) [2, 3, 8, 9, 12, 25–33]. On the Qinghai-Tibet Plateau, where echinococcosis remains prevalent, *E. granulosus* (*s.s.*) has been found in humans, sheep, yaks, cattle, dogs and Tibetan pigs [13, 15, 17–20, 48–51], and *E. canadensis* (G6) has been found in cattle, camels, yaks, goats and dogs [52–54]. Despite this, to our knowledge there has never been a report of *E. canadensis* (G10). Therefore, in the present study, we confirmed for the first time that yak can also serve as the intermediate host of *E. canadensis* (G10) and also observed some protoscolices in the cyst. However, the true transmission pattern of this genotype needs to be determined by further epidemiological and molecular investigations of animals as definitive and intermediate hosts in the Qinghai-Tibet Plateau. The identification of the G10 genotype of *E. canadensis* in the yak in China shows that this genotype possibly has a wider geographical distribution and broad host range than expected.

There are many domestic yaks and some wild yaks on the Tibetan plateau, which provide a great possibility for the spread of *E. canadensis* (G10). Approximately 22,000 wild yaks live in China, accounting for 90% of the world’s total population [44]. These wild yaks are mainly distributed on the Tibet Plateau [44, 55–59], but it is unclear whether the present finding in a yak was a spillover from a wildlife-cycle [60]. Our study is not only a warning for native people to be aware of the disease, but also has significance for the study of *E. canadensis* (G10) globally. Further studies are necessary to determine host range and specificity, geographical distribution, transmission dynamics, infectivity to animal and humans, etc. of this genotype in China.

## Conclusions

We have confirmed for the first time globally that *E. canadensis* (G10) can use yaks as the intermediate host and form fertile cysts containing protoscoleces in yaks. This suggests that the G10 genotype have a wider geographical distribution and broader host range than previously believed and reported, and that this genotype pose potential risks to human health and animal husbandry. Therefore, our study has important significance for further studying *E. canadensis* (G10) across the world.

## Abbreviations

CAAS: Chinese Academy of Agricultural Sciences
CE: Cystic echinococcosis
DNA: Deoxyribonucleic acid
NTD: Neglected tropical disease
PCR: Polymerase chain reaction
SNR: Short non-coding region
WHO: World Health Organisation
